# A Simple and Fast Manual Centrifuge to Spin Solutions in 96-Well PCR Plates

**DOI:** 10.3390/mps3020041

**Published:** 2020-05-25

**Authors:** Ken Motohashi

**Affiliations:** 1 Department of Frontier Life Sciences, Faculty of Life Sciences, Kyoto Sangyo University, Kamigamo Motoyama, Kita-ku, Kyoto 603-8047, Japan; motohas@cc.kyoto-su.ac.jp; Fax: +81-75-705-1914; 2 Center for Plant Sciences, Kyoto Sangyo University, Kamigamo Motoyama, Kita-Ku, Kyoto 603-8047, Japan

**Keywords:** centrifuge, 0.2-mL 96-well PCR plate, high-throughput screening, PCR solution, spin-down

## Abstract

A simple and fast manual centrifuge was developed to spin down solutions in 96-well polymerase chain reaction (PCR) plates. A commercially available salad spinner was utilized for this purpose. Acceleration and deceleration of the centrifuge were faster than those of a conventional electric centrifuge using 96-well PCR plates. Solutions in a 96-well PCR plate settled quickly after centrifuging for only 3 s. This lightweight centrifuge can be stored under a laboratory bench or on a shelf and can be put on the bench only when required, whereas the electric centrifuge is immobile due to its weight and the requirement of electric cables. This simple centrifuge is inexpensive, requires minimal effort for making, and can be used anywhere.

## 1. Introduction

Genotyping by polymerase chain reaction (PCR) is an important technique for screening mutants in various organisms [1,2,3] and genome editing by CRISPR/Cas9 [4,5,6,7]. Mutant screening by PCR is efficient for multiple samples. *Escherichia coli* colony-PCR is also an efficient technique for screening positive colonies [8,9,10], especially for high-throughput-cloned *E. coli* plasmids expressing recombinant proteins [11]. Microfluidic devices have recently been coupled with PCR as a method for high-throughput PCR screening [12]. However, conventional PCR using plasticware (0.2-mL 96-well PCR plates) are also widely used in high-throughput PCR screening. Since screening reactions include small volumes of PCR mixtures (~10 µL), the solution is required to be spun down before agarose gel electrophoresis [13,14,15]. Several commercially available electric centrifuges can be used for this purpose. However, these centrifuges require a few seconds for acceleration and deceleration to spin the PCR solutions down in 96-well PCR plates. Moreover, the machines require electricity and occupy significant amounts of space on laboratory benches.

A commercially available salad spinner can be used as a low-cost alternative to cytocentrifuges [16,17]. The basket in the salad spinner can rotate at ~600 revolutions per minute (rpm) [16]; this speed of rotation and centrifugal force is sufficient for hematocyte preparations [16,17]. Recently, a high-speed hand-powered paper centrifuge was developed for separation of plasma from whole blood [18]. The paper centrifuge can simultaneously process eight samples with high centrifugal forces of 30,000× *g*. In this study, I developed a manual centrifuge to spin solutions down in 96-well PCR plates for conventional PCR screening, thereby significantly improving the commercially available salad spinner. The manual centrifuge is lightweight, portable, and usable in any space.

## 2. Experimental Design

A manual centrifuge was developed to spin solutions in 96-well PCR plates with a base to fix two 96-well PCR plates that can be centrifuged simultaneously. The improved salad spinner can be used as a simple centrifuge machine with a speed of ~700 rpm and functions efficiently with 96-well PCR plates for a short spin. A conventional electric centrifuge was used to compare the characteristics of the manual centrifuge.

### 2.1. Materials

Polystyrene foam (200 × 200 mm, thickness 25 mm) was procured from the lid of a reagent delivery box and used as the base for fixing two 96-well PCR plates. A salad spinner (Pearl Metal Co., Ltd., Sanjo, Japan; Cat. No.; Petit chef Jr C-750, http://www.kitchen-tool.com/goods_C-750.html, https://amzn.to/2AkQcN0) was used as the body of the manual centrifuge to spin solutions down in 96-well PCR plates. A semi-skirted 0.2-mL 96-well PCR plate (Nippon Genetics, Tokyo, Japan; Cat. no.; 4Ti-0760) was used to spin solutions down with the manual and electric centrifuges. EZ-Vision One DNA 6× loading buffer (VWR Life Science, Radnor, PA, USA; Cat. No.; N472-KIT) was used to stain DNA after agarose gel electrophoresis [19].

### 2.2. Making the Manual Centrifuge for Spinning down Solutions in 96-Well PCR Plates (Time for Completion: ~30 min)

#### 2.2.1. Processing Polystyrene Foam for the Base to Fix Two 96-Well PCR Plates

**Figure 1:**
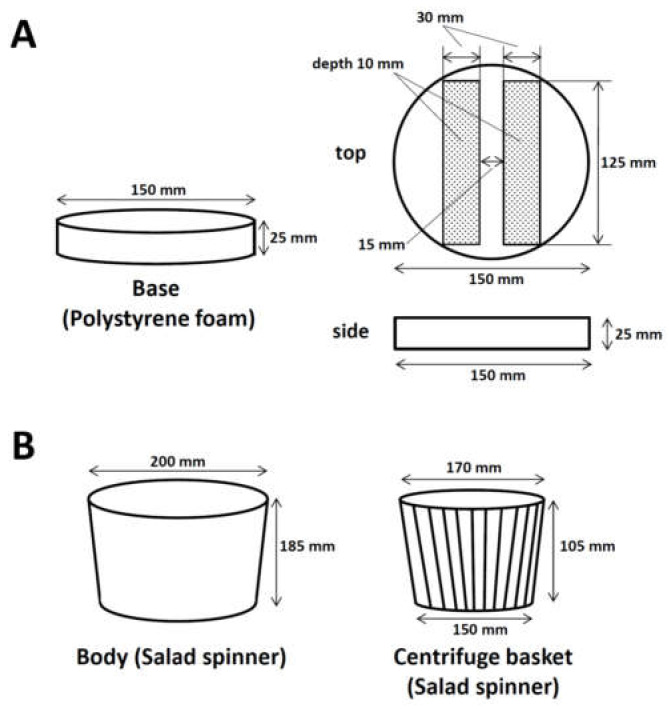
Design of the manual centrifuge for 96-well PCR plates. (**A**) The base to fix two 96-well PCR plates during centrifugation. Polystyrene foam was cut into a 150-mm diameter circle (left). The upper surface of polystyrene foam (hatched area) was scraped out at 10 mm depth (right) as a base to fix the two 96-well PCR plates. (**B**) Body (left) and centrifuge basket (right) of salad spinner.

**Figure 2:**
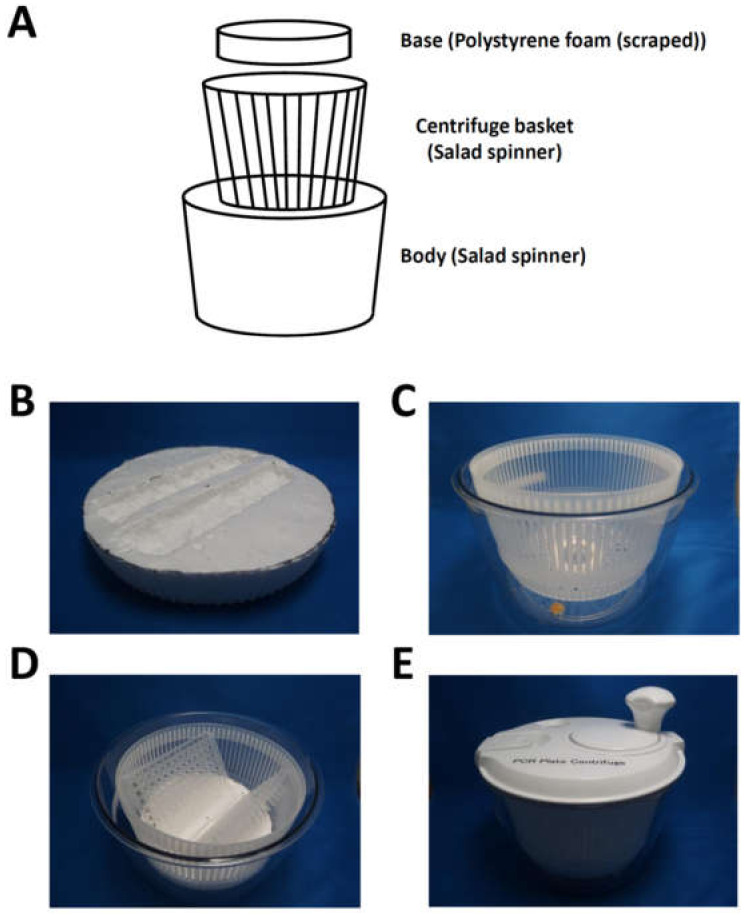
Assembly of the manual centrifuge for two 96-well PCR plates. (**A**) Processed polystyrene foam base as placed into the bottom of the salad spinner. (**B**–**E**) Manual centrifuge for 96-well PCR plates. A base made of polystyrene foam (**B**) and parts of the salad spinner (**C**) were assembled to fix and centrifuge two 96-well PCR plates (**D**). The complete assembled manual centrifuge (**E**).

A circle of diameter 150 mm was cut out of the polystyrene foam (Figure 1A left) and the upper surface of the foam was scraped (hatched area) to a depth of 10 mm using a cutter knife (Figure 1A (right) and Figure 2B) to fix two 96-well PCR plates. The base of the processed polystyrene foam was placed at the bottom of the salad spinner (Figure 1B and Figure 2A).

#### 2.2.2. Assembly of the Manual Centrifuge

The processed polystyrene foam base was assembled with the body and centrifuge basket (Figure 2A). Figure 2B–E show the parts and assembled centrifuge used to spin solutions down in the 96-well PCR plates.

### 2.3. Equipment

The MPS 1000 Mini Plate Spinner Centrifuge (Labnet International, Inc., Cary, NC, USA; https://www.labnetinternational.com/products/laboratory-centrifuges/mini-centrifuges/mps-1000-mini-pcr-plate-spinner) was used as the control electric centrifuge to spin solutions down in 96-well PCR plates in comparison with the manual centrifuge developed in this study.

## 3. Procedure

### Centrifuging Solutions in 96-Well PCR Plates before DNA Agarose Gel Electrophoresis (Time for Completion: ~5 min)

1. Add 2 µL of EZ-Vision One to all PCR samples (10 µL) in 96-well PCR plates.
2. Set two 96-well PCR plates on the base of the manual centrifuge (Figure 2D). 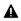 **CRITICAL STEP:** When centrifuging one 96-well PCR plate, another should be placed on the opposite side for balance.
3. Rotate the handle of the salad spinner for 3 s.
4. Check that the PCR mixtures have settled to the bottom of the wells in the 96-well PCR plate and load samples for agarose gel electrophoresis [19]. **OPTIONAL STEP:** The centrifuge can also be used to spin the PCR reaction mixtures down in 96-well PCR plates before PCR.

## 4. Expected Results

**Figure 3:**
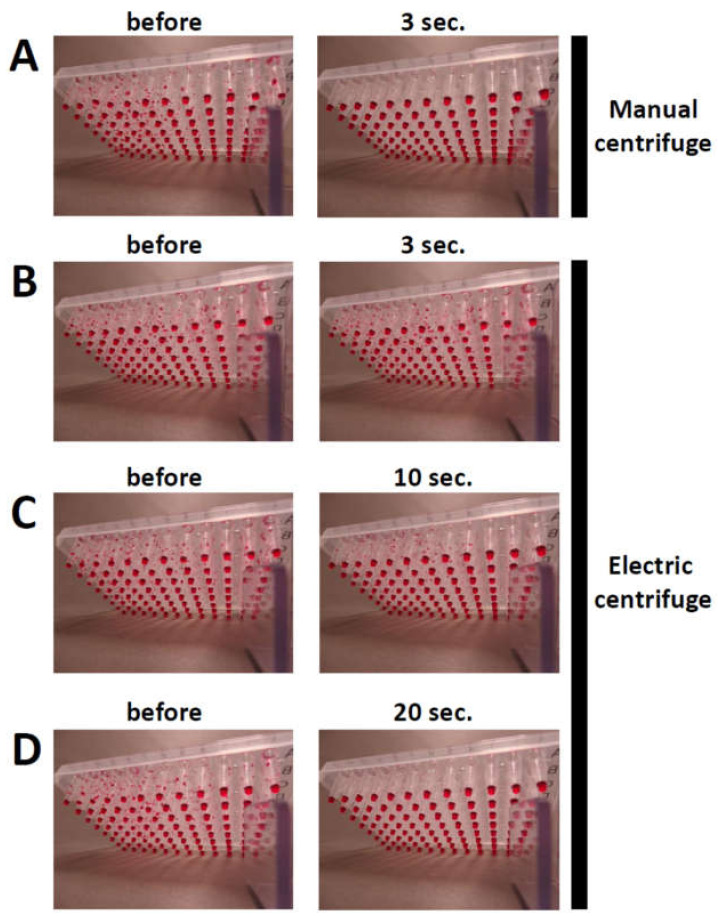
Centrifuged PCR solutions in 96-well PCR plates. The efficiency of centrifuging the PCR solutions was evaluated before (left) and after (right) spinning. Centrifugation was performed for the indicated times (right; the times include start and stop of rotation). Manual centrifuge (**A**). Electric centrifuge (**B**–**D**).

**Figure 4:**
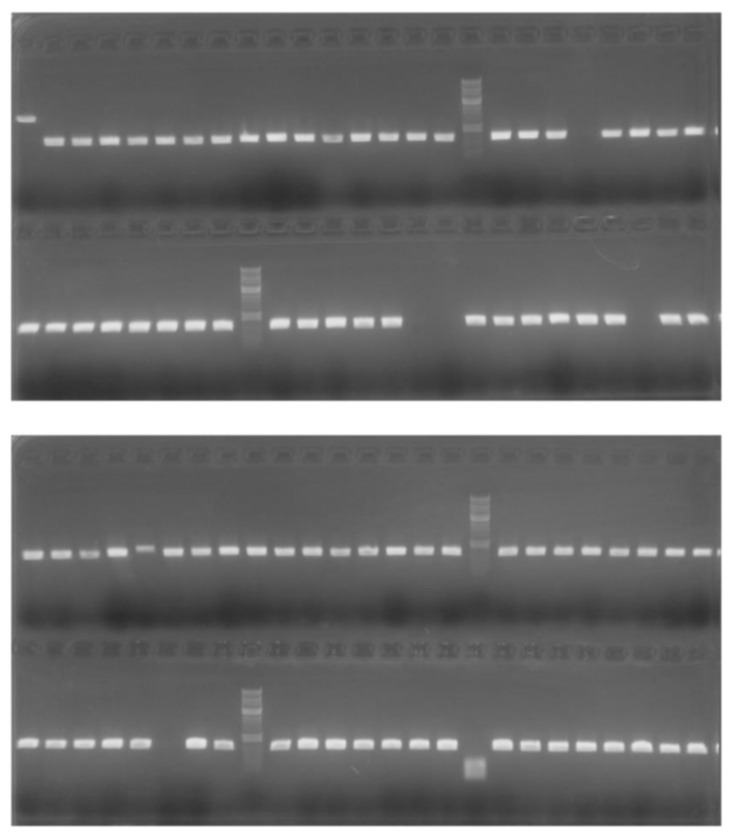
Analysis of spun-down PCR samples by DNA agarose gel electrophoresis. The colony-PCR samples for positive clone screening were stained with EZ-Vision One DNA 6× loading buffer, separated by 1.2% agarose gel electrophoresis [20], and visualized by a combination of a black light and a longpass emission-filter (SC-46, Fujifilm, Tokyo, Japan) [19].

PCR solutions in 96-well PCR plates were sufficiently centrifuged using the manual centrifuge for 3 s (Figure 3A). DNA fragments from these centrifuged PCR mixtures could be clearly visualized by DNA agarose gel electrophoresis (Figure 4) [19]. 

In contrast, using the conventional electric centrifuge resulted in poor centrifugation after 3 and 10 s (Figure 3B,C); this could be attributed to the long time required for acceleration and deceleration. Centrifugation for 20 s by the electric centrifuge was necessary to sufficiently spin the 96-well PCR plates (Figure 3D). In contrast to the manual centrifuge, the electric centrifuge has a heavy metal drive system for centrifugal forces higher than 500× *g*, which is associated with a longer duration of acceleration and deceleration during centrifugation. The manual centrifuge developed in this study is a light plasticware that can accelerate and decelerate quickly to help the mixtures with settling down. Therefore, although the manual centrifuge did not rotate at a high speed or centrifugal force, 3 s was enough to spin the solutions down in the 96-well PCR plates. The manual centrifuge using a commercially available salad spinner was previously developed with a relative centrifugal force of 24× *g* for centrifugation of blood samples (Table 1) [16,17]. The manual centrifuge developed in this study was modified to centrifuge solutions in 96-well PCR plates and its centrifuge force (25× *g*) was similar to that previously reported [16]. Although the manual centrifuge developed in this study has to be constructed by the user, its construction is simple and takes a short time (~30 min). The polystyrene foam base was simply cut out and set into the bottom of the salad spinner to fit two 96-well PCR plates.

Table 2 shows the features of the two centrifuges for the 96-well PCR plates. The conventional electric centrifuge for 96-well PCR plates had insufficient acceleration and deceleration and required a few tens of seconds to spin the solutions down in the 96-well PCR plates. Moreover, the electric centrifuge had a built-in electric motor, which made it heavy owing to the presence of multiple components. Therefore, the machine was hard to move and constantly occupied a significant amount of space on the laboratory bench. In contrast, the manual machine developed in this study was light (~0.59 kg) and could be carried with one hand. When not needed, the machine could be stowed under a laboratory bench or on a shelf. Recent research utilizes a wide range of techniques from molecular biology to biochemistry. Thus, the efficient use of small laboratory space for successfully performing a variety of experiments requires equipment to be easily portable.

The manual centrifuge was associated with quick acceleration and deceleration and sufficiently spun the solutions in the two 96-well PCR plates after 3 s. Operation of the manual centrifuge did not require electricity, and the instrument functioned on a simple mechanism, and its operation was effortless. Moreover, the price of the salad spinner for the body of the manual centrifuge was notably less expensive than an electric centrifuge, costing only ~USD 18 (i.e., 1/40th of the cost of one electric centrifuge). Thus, the manual centrifuge developed in this study was simple, fast, inexpensive, and useful for spinning solutions down in 96-well PCR plates.

## Figures and Tables

**Table 1:** Features of hand-powered manual centrifuges for various applications.

	Manual Centrifuge for the 96-Well PCR Plates	Manual Centrifuge for the Blood Sample	Paper Centrifuge for the Blood Sample
Application	Spin-down for PCR solution	Separation of plasma for whole blood	Separation of plasma for whole blood
Sample Capacity	96 samples × 2	30 samples	8 samples
Materials	Salad spinner	Salad spinner	Paper and string
Maximum Speed	760 rpm (25× g)	600 rpm (24× g)	125,000 rpm (30,000× g)
Price	USD 18	USD 35	USD 0.2
Weight	0.59 kg	1.5 kg	2 g
Reference	This study	[16]	[18]

**Table 2:** Features of manual and electric centrifuges for 96-well PCR plates.

	Manual Centrifuge (Salad Spinner)	Electric Centrifuge (Labnet MPS1000)
Time of Centrifugation	3 s	20 s
Sample Capacity	Two 96-well PCR plates	Two 96-well PCR plates
Maximum Speed	760 rpm (25× *g*)	2500 rpm (500× *g*)
Price	USD 18	USD 710
Power Supply	None	~120 V, 50/60 Hz
Weight	0.59 kg	1.5 kg
Installation Area	205 × 200 mm (mobile)	190 × 210 mm (immobile)
